# Smoldering mantle cell lymphoma

**DOI:** 10.1186/s13046-017-0652-8

**Published:** 2017-12-15

**Authors:** Haige Ye, Aakash Desai, Dongfeng Zeng, Krystle Nomie, Jorge Romaguera, Makhdum Ahmed, Michael L. Wang

**Affiliations:** 1 Department of Hematology, the First Affiliated Hospital of Wenzhou Medical University, Wenzhou, China; 2 Department of Lymphoma/Myeloma, The University of Texas MD Anderson Cancer Center, 1515 Holcombe Blvd, Houston, TX 77030 USA; 3 University of Texas Health Science Center at Houston, Houston, TX USA

**Keywords:** Mantle cell lymphoma, Indolent MCL, Smoldering lymphoma

## Abstract

**Background:**

Mantle cell lymphoma (MCL) is an aggressive disease, with poor prognosis and a limited survival. However, some patients with indolent MCL can survive beyond 7~10 years. These patients remain largely asymptomatic and can be in observation for a long time without any treatment. The process of “wait and watch” leaves these patients with the potential risk of evolution to classic, aggressive MCL. On the other hand, early treatment for these patients may not impact overall survival but rather affects the quality of life. Therefore, it is essential to clearly identify this type of indolent MCL at the time of diagnosis.

**Results:**

Reported findings of indolent presentation of MCL include: lack of B symptoms, normal serum lactic dehydrogenase (LDH) and β2-microglobulin levels (β2M), low MCL-International Prognostic Index (MIPI) score, maximum tumor diameter less than 3 cm, spleen size < 20 cm, positron emission tomography/computerized tomography with the Standard Uptake Value max <6, Ki-67 less than 30%, with some particular immunophenotype, such as CD5 and CD38 negative, markedly increased CD23 positive lymphocytes proportions, high expression of CD200, kappa light chain restriction, without *C-myc*, *TP53* and *NOTCH1/2* mutations, non-blastoid/pleomorphic histology, and no tumor growth on reevaluation every 2~3 months (followed for at least 6 months). Imaging evaluation may only be performed in the presence of disease-related symptoms or organ involvement. Meanwhile, if novel nodal or extranodal lesion is found, biopsy is mandatory to exclude lymphoma.

Common clinopathological forms of indolent presentations include monoclonal B lymphocytosis with t (11; 14); “indolent leukemic” presentation of MCL with involvement of peripheral blood, bone marrow involvement, splenomegaly, and minimal lymphadenopathies and in situ lymphoma (often found in lymph nodes removed for other reasons, and in gastrointestinal biopsies).

**Conclusions:**

Considering these distinct indolent clinical presentations with particular features in cytology and gene mutational status, we propose to include these MCL clinical presentations under the umbrella of “Smoldering Mantle Cell Lymphoma”.

## Background

Mantle cell lymphoma (MCL) is an aggressive disease, with a poor prognosis and limited survival [1]. However, up to 30% of MCL patients may have an indolent clinical course [2, 3], with survival exceeding 7~10 years [4]. These asymptomatic patients can survive despite no initial treatment using the “wait-and-watch” approach. The clear ability to differentiate between the aggressive and indolent MCL types is necessary to guide and choose which therapeutic approach should be administered. However, no established criteria are currently used to inform the treatment decision-making process for MCL patients with the indolent clinical course. Consequently, criteria for indolent MCL would help identify patients who are at risk of early progression and who are in need of immediate therapy, sparing patients from toxic treatment.

The classification and nomenclature of such indolent MCL cases remain unclear. The terms “classical MCL”, “subclinical disease”, “preclinical phase” or even “benign lymphoma” have been used previously [5]. Another report coined the term “non-nodal type of MCL” or “indolent leukemic presentation” [6]. We propose to unify all of the indolent MCL presentations under the title of “Smoldering Mantle Cell Lymphoma (SMCL)” to emphasize its initial indolent behavior and its predisposition to develop into a more clinically aggressive lymphoma. We propose that the following parameters can be used to define SMCL: lack of B symptoms, normal serum lactic dehydrogenase and β_2_-microglobulin levels; low MCL-International Prognostic Index (MIPI) score, maximum tumor diameter < 3 cm, spleen size <20 cm, Positron Emission Tomography/computerized tomography (PET/CT) with the Standard Uptake Value (SUV) max <6; Ki-67 < 30%; absence of *c-myc*, *TP53* and *NOTCH1/2* mutations, non-blastoid/pleomorphic histology; and no tumor growth on re-evaluation every 2~3 months (followed for at least 6 months). Imaging evaluation may only be performed in the presence of disease-related symptoms or organ involvement. If novel nodal or extranodal lesion is found, biopsy is mandatory to exclude lymphoma. Meanwhile, the full understanding of this definition will evolve as the genetics and heterogeneity underlying MCL progression are better understood and as potential biomarkers are uncovered. Furthermore, in this molecular era with the advent of big data and detailed DNA/RNA/proteomics analysis, more precise guidance regarding this classification will be available in the foreseeable future.

## Proposed clinical features of SMCL (Table 1)

### MIPI, Ki-67 proliferation marker

In 2008, the MIPI scoring criteria were established to enable the stratification of clinically diverse MCL patients into three risk groups: low risk, intermediate risk and high risk The MIPI criteria include the following 4 independent pre-treatment prognostic factors: age, Eastern Cooperative Oncology Group performance status, pre-treatment ratio of actual/upper limits of normal for lactate dehydrogenase (LDH), and pre-treatment white blood cell (WBC) counts [7]. In this report, patients with a score of 3 or less were said to have indolent disease and could defer therapy for a median of 1 year. Moreover, once these patients began therapy, the patients showed more sensitivity to treatment, with a 5-year median survival of 60%. This risk model has been validated in other retrospective and prospective clinical publications [8, 9]. In addition to these variables, tumor proliferation is recognized as one of the strongest biological prognostic factors in MCL, and high tumor cell proliferation indicates shorter survival [10]. In order to predict MCL survival, a mathematical model was established [11]. The quantitative model combining 20 different expression levels of proliferation genes in MCL was used to determine the extent of proliferation. The proliferation signature average was inversely correlated with survival with high statistical significance not only in the training set (*p* = 1.92X10^−5^) but also in the validation set (*p* = 7.44 X10^−5^). Additionally, this signature average was correlated with the number of Ki67-positive cells (*r* = 0.69), which is an immunohistochemistry (IHC) marker of proliferative index. The IHC Ki-67 data is not as strong but is readily available in clinical practice and also correlated with OS [12, 13]. Hoster et al. reported that significant differences in time to treatment failure (64 months versus 19 months) and overall survival (OS) (not reached versus 45 months) were found between the Ki-67 level < 30% group versus the Ki-67 level ≥ 30% group in 543 MCL cases (*p* < 0.0001 each) [12]. Later, the Ki-67 index and MIPI were combined and modified to create the MIPI-c. This newer scoring criteria separated four groups of 508 patients with the following varying 5-year OS rates: 85, 72, 43, and 17% (*P* < .001) and was more discriminative than the MIPI criteria alone [14]. We believe that the MIPI-c scoring system should be used to diagnose SMCL, with SMCL scored as a low MIPI score ≤ 3 and a Ki-67 index <30%.

**Table 1:** Comparison of clinical, morphology, immunophenotype and genetics features between SMCL and classic MCL

	SMCL	Classic MCL
Clinical features		
B symptom	without	with or without
Serum LDH	normal	elevated or normal
Serum β2-MG	normal	elevated or normal
MIPI	low risk	low to high risk
Ki-67	< 30%	≥30%
Max tumor diameter	< 3 cm	≥3 cm
SUV of PET/CT	≤ 6	> 6
evaluation every 3 mons	no tumor growth	having tumor growth
Morphology		
Origin	germinal or post-germinal center	antigen-naïve pregerminal center
Cytology	non-blastoid/pleomorphic	small cell, classic, pleomorphic, or blastic
Immunophenotype		
CD5	low	high
CD38	low	high
CD23	high	low
CD200	high	low
light chain restriction	kappa	lambda
Genetics		
*TP53*	low	high
*NOTCH1/2*	low	high
*C-myc*	low	high
Cyclin D1 mRNA	low	high
Cell cycle	G1 or before	S

*MCL* mantle cell lymphoma, *LDH* lactic dehydrogenase, *β2-MG* β2-microglobulin, *MIPI* mantle cell lymphoma International prognostic index, *Max* maximum, *SUV* standardized uptake value, *PET/CT* positron emission tomography/computerized tomography

### Tumor size

According to the Goldie-Coldman hypothesis, the probability that a cancer contains drug-resistant clones depends on the size of the tumor and the mutation rate. Larger tumor masses have a more heterogeneous tumor cell population, which can lead to chemotherapy resistance [15]. Additionally, decreased vascularity in the center of larger tumor masses may result in less exposure to chemotherapeutic agents. Several studies have suggested that larger tumor size acts an adverse prognostic factor [16–18], and various studies have shown tumor sizes can range from 5~10 cm. In other reports, patients with a higher tumor volume had inferior complete remission rates and higher relapse rates [19, 20]. In patients with stage 1 and 2 tumors who only received radiotherapy, one study found that patients with maximum tumor diameter < 3 cm had a local recurrence rate of 5% compared with 18% for those patients with a maximum tumor diameter ≥ 3 cm [21]. Thus, we define the threshold for tumor size at 3 cm for patients in early stage of the disease showing indolent clinical behavior in SMCL. This definition of <3 cm stems primarily from clinical intuition and over 20 years in the clinic rather than statistical data.

### Spleen size

Spleen size is significantly influenced by body height and sex [22]. Splenomegaly occurs frequently in MCL patients, with a spleen measured beyond 15 cm in its longest dimension considered enlarged, and spleen size larger than 20 cm considered significant. Here, we define the upper threshold of enlargement of spleen size in SMCL as 20 cm. If spleen size is less than 20 cm, and the patient is without symptoms related to the enlarged spleen such as shortness of breath, early satiety, gastric reflux, walking problems and left upper quadrant pain, no treatment is necessary. We emphasize that an enlarged spleen may cause cytopenia, with the patient at risk for spontaneous rupture and infarction [23, 24]; therefore, we recommend that MCL patients with spleen size above 20 cm should undergo treatment.

### PET/CT standard SUV

Previous data showed that low SUV observed with PET/CT correlated with an indolent clinical process [25–27]. In a retrospective study, MCL patients with a PET SUVmax <5 had better OS and FFS (87.7% vs. 34%, 45.3 m vs. 10.6 m, *P* < 0.01 and <0.001, respectively) [26]. Other studies have suggested that SUV combined with IPI can enhance the stratification and predict prognosis at the time of diagnosis [27]. MCL patients with IPI ≤ 2 and SUVmax ≤6 (low-risk, *n* = 9, 29%) were found to have the best event-free survival (EFS) compared to those with IPI > 2 or SUVmax >6 (intermediate-risk group, *n* = 13, 42%), IPI > 2 and SUVmax >6 (high risk, *n* = 9, 29%). At a median follow-up of 21 months, no relapse has been observed in the low-risk group, while the median EFS durations in the intermediate- and the high-risk groups were 37 and 22 months, respectively (*p* = .004) [27]. We propose, based on the latter, to include this PET value in the definition of SMCL at a SUVmax <6 with PET/CT.

Therefore, the clinical characteristics of SMCL should include the following: low MIPI scores, Ki-67 < 30%, maximum tumor diameter less than 3 cm and low SUV of PET/CT. In addition, we believe that the SMCL diagnostic factors should also include a lack of B symptoms [28], normal serum LDH [7, 29] and β2-microglobulin levels [28, 30], spleen size <20 cm and no tumor growth on reevaluation every 3 months (followed up for at least 6 months) [28].

## Biologic markers

### Cytology

MCL has the following 4 cytological classifications: classic, small-cell, pleomorphic, or blastic [1, 31]. Blastoid or pleomorphic variants are generally characterized by a more aggressive clinical course, higher proliferation rate and worse prognosis [32–35]. Meanwhile, the blastoid morphology is associated with *TP53* mutations, *c-myc* gene abnormalities [36, 37], complex karyotype, and high cyclin D1 mRNA levels [38], which are all indicative of high proliferation and inferior survival [38]. The median OS for MCL patients with the blastoid variant has been reported at only 11–14.5 months [39, 40]. Additionally, the median survival time for patients whose MCL transformed from nodular or diffuse to blastoid was only 3.8 months [95% Confidence Interval (CI): 2.4–5.2 months] as compared with 26 months following the latest rebiopsy in patients without transformation (95% CI: 17–35 months, *P* < 0.001) [39]. The pleomorphic variant of MCL is composed of numerous large cells with irregular nuclear contours and prominent nucleoli [41], mimicking diffuse large B-cell lymphoma [42], which is also considered as an aggressive variant with *c-myc* gene mutations [43].

Thus, we conclude that SMCL should be the non-pleomorphic/blastic variants. Except regarding cytology, here, we would not dwell on histology because all 3 clinical presentations of SMCL have no nodes, which we can assess architecture to label the presentation as mantle zone or follicular or diffuse.

### Immunophenotype

In order to explore the difference in immunophenotypic behavior between the indolent MCL and typical MCL, Espinet B, et al. measured the expression of CD38/CD200 by cytometry in non-nodal cases with cyclin D1–positive monoclonal asymptomatic lymphocytosis (MALD1) and typical MCL [44]. The B cells showed a significantly higher expression of CD38 in 24 cases of typical MCL. (median, 89%; range, 0–100%), compared to that of the 13 cases of MALD1 (median, 14%; range 0–35%). Low or absent expression of CD200 in B cells was found in 15 cases of typical MCL whereas higher expressions of CD200 positive B cells were detected in the 12 cases of MALD1. These results were also confirmed by qRT-PCR analysis. Thus, CD38 combined with CD200 could be helpful to differentiate MBL with t (11;14) from typical MCL.

High expression of CD38 in typical MCL suggest that CD38 may contribute to the survival of B cell neoplasm via adhesion molecules such as CD31 [45]. Low expression of CD38 in MALD1 may weaken the interaction between MALD1 B cells and micro vessels with CD31 expression, which result in reduced accumulation of clonal B cells. Expression of CD200 is decreased in all typical MCL, while it is highly expressed in most MALD1. CD200 binding to its receptor CD200R may produce inhibitory signals, which can cause reduced B cell proliferation. This is thought to play a crucial role of preventing overproduction of tumor cells and maintaining asymptomatic condition over a long period of time in MALD1. Conversely, with decreased expression of CD200, inhibitory signals for B cells are lost which causes uncontrolled B cell proliferation leading to the transformation of an indolent condition to an aggressive one [46].

In addition, the CD5 of a typical MCL is usually positive, however, a small subgroup of patients with MCL may have a negative CD5. The peripheral blood of such patients has atypical lymph cells, with or without lymphocytosis or lymphadenopathy, or splenomegaly, but with indolent clinical process [47].

### Genetics

In the pathogenesis of MCL, CCND1 appears to be a weak oncogene. Therefore, the clinical aggressiveness of MCL is possibly associated with a secondary abnormal cytogenetic event. MCL has a highly unstable genome, which may lead to recurrent abnormalities, including loss of chromosome 1p, 8p, 9p (*CDKN2A, CDKN2B*), 9q, 11q (*ATM*), 13q14 and 17p (*TP53*) or addition of 3q, 8q (*MYC*), 10p (*BMI1*), 15q, and 18q. These mutations may cause damage to DNA repair [48] and contribute to hyperproliferative mutations [49, 50]. Although IGH/CCND1 rearrangement is the most crucial initial event, additional gene mutations or the addition of aberrant cytogenetic event are necessary to destabilize the indolent lymphoma. A previous study demonstrated that the genes involved in this destabilization process may be INK4a/CDK4/RB1, ARF/MDM2/p53, Cyclin D1/cdk4 (6) kinases, cyclin E/cdk2 kinase and p16INK4a, and others [11, 51, 52]. However, the mechanism is generally complicated, which needs to be further elucidated. Moreover, the time necessary for the accumulation of sufficient lesions to develop classic MCL remains unknown.

### IGVH mutational status

MCL originates from antigen-naïve pre-germinal center B cells located in the mantle zone surrounding the germinal center [1, 53]. Cells from the mantle zone display high clonal diversity and express germline Ig heavy chain (IgH) V genes [54]. In contrast, in the germinal center, few B-cell clones have intraclonal diversity via somatic hypermutations in the VH region genes. MCL generally expresses VH genes, without any or minimal somatic mutations. Of particular importance, indolent MCL (70%) demonstrates significantly higher hypermutated immunoglobulin gene rearrangements compared with classical MCL (~20%) [55], suggesting that indolent MCL has germinal center or post-germinal center origin [56]. Orchard et al. found that *IGVH* gene mutation rates in leukemic MCL are much higher than that in nodal MCL among the 80 cases of MCL studied [57]. However, the clinical significance of *IGVH* gene rearrangement in MCL remains dubious. Most studies have shown no correlation with IGVH rearrangement with the outcome of the patients [58–60], in other cases, longer survival in MCL with IGVH rearrangement has been observed [61–63]. Therefore, *IGVH* gene mutation status is not included in the definition of SMCL.

### SOX-11

In classic aggressive MCL, the neural transcription factor *SOX11* gene is aberrantly expressed [56]. The prognostic role of *SOX11* have shown conflicting results. *SOX11* was correlated with improved survival, which was reported in two studies with cohorts of 53 and 186 MCL patients, respectively [55, 64–66]. In contrast, a negative correlation between *SOX11* and better survival was reproted in two other series [55, 66]. In the recent Nordic MCL data, *SOX11* was proposed to be routinely assessed with *TP53* and patients with high *SOX11* expression had superior OS and EFS compared to patients with low *SOX11* expression [8]. However, at Lugano conference in 2017, *SOX11* has no prognostic value in MCL [67]. Thus, *SOX11* is not included in the definition of SMCL.

### TP-53

TP53 dysfunction gives rise to mutant protein that affects the generation, development and progression of diffuse large B cell lymphoma. In essence, TP53 mutations lead to irregular B-cell phenotypes and are associated with poor overall survival [68]. TP53 has also been shown to be associated with significant independent molecular markers that are correlated with dismal outcomes [38, 69–71]. Furthermore, as demonstrated at Lugano conference in 2017, TP53 but not SOX11 immunohistochemistry has prognostic value independent of MIPI and Ki-67 [67]. In this study, MCL patient samples were sorted into four categories according to different percentage of P53 immunohistochemistry staining (0%, 1–10%, 11–50% and >50%). The results showed that TP53 deletion (0%) had no significant influence on the TTF and OS of MCL patients (*p* = 0.10, HR = 1.45 and *p* = 0.18, HR = 1.46, respectively), while TP53 mutations (>50%) are highly predictive for short time to treat failure (TTF) and poor OS (both *p* < 0.0001; HR 2.47 and 3.00).

Therefore, the *TP53* mutational status needs to be routinely tested to define and diagnose SMCL.

### NOTCH 1/2

*NOTCH1/2* mutations accounted for 5~12% of MCL [72, 73]. The deregulation of *NOTCH* pathway is implicated in developmental disorders and oncogenesis [72]. In MCL cell lines, the inhibition of the *NOTCH* pathway resulted in reduced proliferation and/or increased apoptosis [72]. *NOTCH1* mutations in MCL patients correlated significantly with poor OS [72]. Bea et al. found that *NOTCH2* mutations are also significantly associated with poor clinical outcome using whole-genome/whole-exome analysis in 39 MCL and subsequent validation in and additional 172 cases [73]. Thus MCL with *NOTCH1/2* mutations should also be rule out from SMCL.

### MYC

The *MYC* oncogene can activate cell growth and cell cycle progression gene transcription [74, 75]. MCL with *MYC* mutations is associated with blastoid /pleomorphic morphology, indicating a poor prognosis [36, 76–78]. In a study of 65 patients with MCL [78], *MYC* mutations were higher in blastoid/pleomorphic MCL variants (mean, 19.0%) than in classic MCL (mean, 1.9%; *P* < 0.001). Also, high *MYC* mutations were significantly associated with higher expression of *p53* and Ki-67 and shortened OS and PFS (all *P* < 0.05). Accordingly, *MYC* mutation is included in the definition of SMCL.

### Genetic profiling

Ideally, a combination of the proliferation gene expression signature with different oncogenes related to the MCL cell cycle could provide further information regarding biological behavior of MCL and better guide its management. As early as 2003, Rosenwald et al. had reported that increased cyclin D1 mRNA expression levels combined with INK4a/ARF locus deletions correlated with increased proliferation rate and shorter survival [11]. The MKI67 gene that encodes the important proliferation marker Ki-67 was shown to be independently prognostic, even with modern therapies [79, 80]. Furthermore, in order to bypass the need for fresh tissue for pathologic analysis, a 17-gene proliferation signature based on the MKI67 gene and analyzed by nanostring in formalin-fixed paraffin-embedded (FFPE) has been reported. This assay (MCL35) assigned patients to high-risk (26%), standard-risk (29%), and low-risk (45%) groups, with median OS of 1.1, 2.6, and 8.6 years, respectively (*p* < .001), and this was independent of MIPI risk assessment.The analytic and clinical validity of this assay provide a reliable biomarker to support risk-adapted clinical trials [81].

## Common clinopathological forms of SMCL (Fig. 1)

### Monoclonal B lymphocytosis (MBL) with t (11;14) (q13; q32) and cyclin D1-positive (MALD1)

With the advent of flow cytometry, monoclonal lymphocytes can be found in up to 3.5~12% of healthy population aged over 40 years old [82–88]. “Monoclonal B-lymphocytosis” (MBL) is defined as the count of B-lymphocytes in the peripheral blood less than 5000/ul [89], with lack lymph node or organ enlargement, cytopenias, or disease-related symptoms [90]. Additional features of MBL include overall kappa: lambda ratio > 3:1 or <0.3:1, or >25% B cells with absent or low expression of surface immunoglobulin or a disease-specific immunophenotype, and clinical stability over a 3-month period at follow-up. Different counts of B cells are usually related to varied clinical conditions. High-count MBL is considered as a pre-neoplastic condition, which may progress into other B cell neoplasms like chronic lymphocytic leukemia (CLL). However, a low-count state of MBL does not usually develop into leukemia, although the disease can be present for many years [91].

**Fig. 1:**
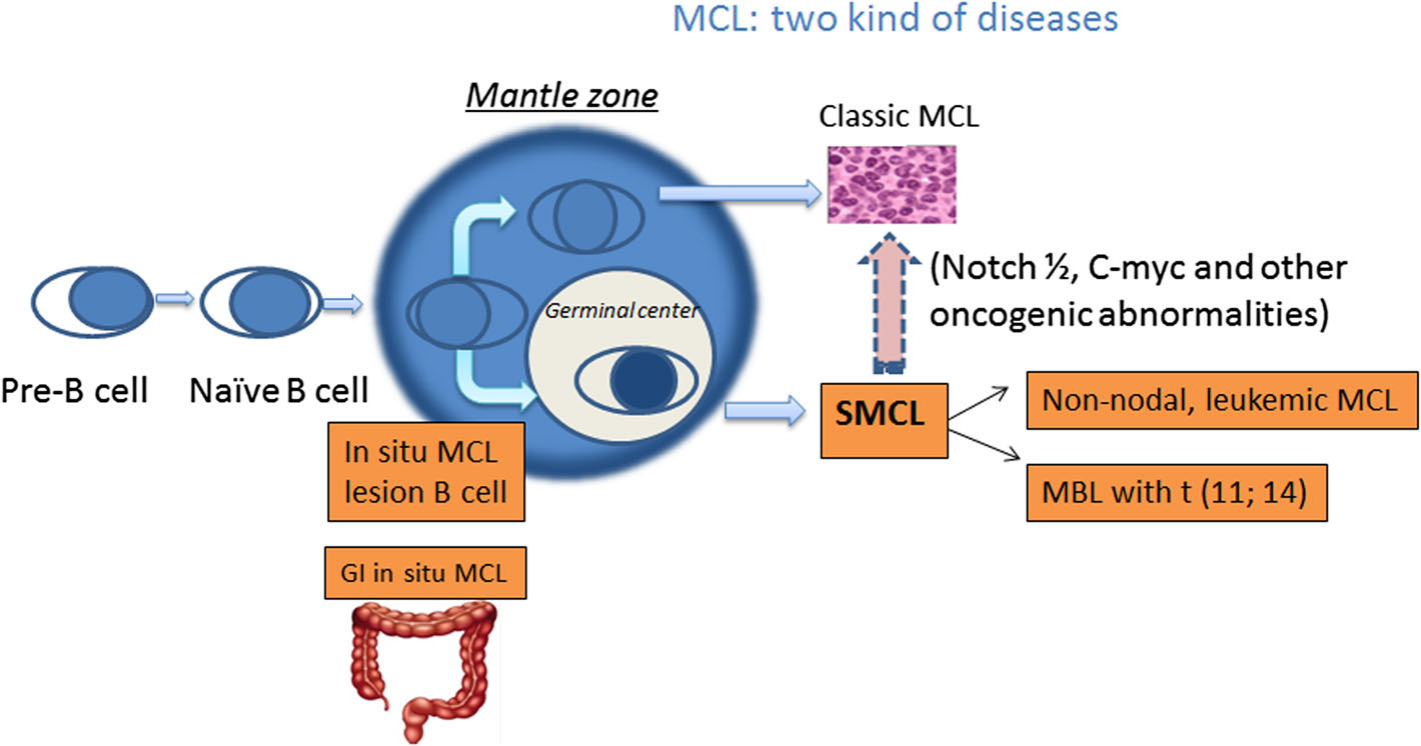
MCL actually can be divided into two kinds of disease entities: classic MCL and SMCL. SMCL includes the following hypothetical models: MBL with t (11; 14), non-nodal leukemic MCL and in situ MCL, especially GI in situ MCL. SMCL, smoldering mantle cell lymphoma; MBL, monoclonal B lymphocytosis; GI, gastrointestinal

Asymptomatic MBL with t (11; 14) (q13; q32) and cyclin D1-positive (MALD1) is very rare, accounting for only 3% of all MCL diagnosed [44, 92]. MALD1 usually without enlargement of the lymph node and spleen, has a very indolent clinical process without treatment, with survival for up to 9 years [44, 47, 55, 87]. MALD1 has a different phenotype and gene expression profile when compared with typical MCL, with a high rate of IGVH gene mutations, lack of genomic complexity, and absence of expression of transcription factors of the high-mobility genes [55]. The gene signature of MALD1/MCL showed CD200 upregulated in MALD1 and CD38 upregulated in MCL [44]. Pathway analysis using the Ingenuity Pathway Analysis tool indicated that the expression of gene sets of the MALD1 is related to immune activation and inflammatory responses, while those of typical MCL are related to neoplastic behavior and cell proliferation [44].

Thus, we propose to classify MALD1 as SMCL. However, the SMCL like asymptomatic MBL with t (11; 14) and Cyclin D1 expression is different from typical MCL in phenotype and gene expression profile, which may have high rate of IGVH gene mutations, lack of genomic complexity, and absence of expression of transcription factors of the high-mobility genes [55].

### Non-nodal, leukemic MCL (CLL-type MCL)

Elevated leukocyte counts in the MIPI of conventional MCL, as a leukemic presentation, is a feature of adverse prognosis [7]. However, mantle cell leukemia may have two different clinical courses: aggressive and indolent [55, 93]. Ondrejka et al. retrospectively analyzed 8 cases of MCL presented as leukemic forms beyond 10 years. These cases were like CLL, with mild lymphocytosis and without lymphadenopathy and splenomegaly. All cases had positive t (11:14) with considerable proportion of CD23 positive cells, and kappa light chain restriction, while typical MCL cells are generally CD23 negative and lambda light chain restricted. These patients had a long-term stable disease or the lymphocyte counts increased very slowly. After a median follow-up of 27 months, 7 patients survived, including 2 which needed some form of treatment.

Orchard et al. identified a higher incidence of mutated IGVH genes in 22 of 34 (66%) leukemic nonnodal MCL cases compared with 3 of 31 (10%) nodal MCL cases. The positive rate of CD38 in the nodal MCL group was significantly higher than that of the leukemic non-nodal group. The clinical manifestations of these leukemic patients were more like the indolent subtype than the nodal MCL patients [57].

These rare asymptomatic cases are akin to classic MCL in respect to immunohistochemistry and cytogenetics. However, these cases presented as leukemic forms, sometimes with splenomegaly, but without abnormal lymph node architecture. Such leukemic MCL cases have shown an indolent clinical course [6, 47, 52, 92], which have other features like:mutated immunoglobulin genes, low CD38 expression, lack of CD5 expression, and a low number of genomic aberrations [55, 57, 66, 92, 94]. So far, it is not clear that these asymptomatic cases are at the early stage of MCL development or a fully developed leukemic form of an MCL of indolent course. Thus, we classify non-nodal, leukemic MCL as SMCL.

### In situ MCL

This new addition in the 2016 revision of the World Health Organization (WHO) classification of lymphoid neoplasms [95] is typically characterized by the presence of cyclin D1+ cells in the inner mantle zones of follicles, and is often found incidentally [96].

In situ MCL is rare and has an indolent clinical behavior [47, 94, 97–100]. Furthermore, the lymph node structure of “in situ MCL” is intact, so it can only be diagnosed by the detection of cyclin D1 with immunohistochemistry analysis [44, 91, 96, 100, 101]. Generally, “in situ MCL” cases are incidentally found by reevaluation of the previous samples of MCL patients. Racke et al. analyzed the “negative” lymph node samples 2–15 years prior to the diagnosis of 7 cases of MCL, and “in situ MCL” were found in all cases [102]. These early lesions occurred 2–86 months prior to the diagnosis of classic lymphoma, which were characterized by small groups of cyclin D1-positive lymphocytes in the mantle zone of lymph node or extra nodal tissue. After a follow-up study of “in situ MCL”, Carvajal-Cuenca A, et al. found that most of patients may not develop into classic MCL until a long period ranging from 1 to 19.5 years without any treatment [96].

“in situ MCL” is very low risk for the development of classic MCL, however, the acquired alterations in the DNA damage response pathway, such as ataxia-telangiectasia mutated (ATM), or cell cycle checkpoint kinase 2 (CHK2) inactivating mutations may facilitate the development of the tumor [53]. In addition, “in situ MCL” need to be differentiated from MCL with a mantle zone pattern and classic MCL, in that “in situ MCL” usually do not need any therapeutic intervention. Therefore, “in situ MCL” can be classified as another subtype of SMCL.

### Gastrointestinal tract in situ MCL

Most patients with MCL have the gastrointestinal (GI) tract involvement, which manifest in a variety of forms, such as multiple lymphomatous polyposis (MLP) or a slight mucosal change [103, 104]. Primary GI involvement of MCL is rare, which accounts for 4 ~ 9% of GI B-cell non-Hodgkin lymphomas [105]. Some patients with primary GI tract MCL may have very indolent clinical process and good survival [106].

Neto, A. G. et al. reported a case of GI tract in situ MCL [107]. A colonoscopic biopsy following bright red blood per rectum showed benign colonic mucosa. Two years later, the patient had ileocolic intussusception related to enlarged lymph nodes and was confirmed as widespread MCL. Reevaluation of initial colonic biopsies showed cyclin D1–positive cells within small lymphoid aggregates, which were confirmed by FISH for t (11; 14). After chemotherapy, the residual disease had positive cyclin D1 staining and FISH t (11; 14) in mantle zone, which was like the initial lesions. Thus, the initial colonic lesions may be an in-situ GI MCL.

However, it is very difficult to find GI in situ lymphoma at the early onset. Histologically, the lymphoid follicular structure of colonic mucosa remains intact or only shows minimal changes due to ubiquitous lymphoid aggregates in GI tract. However, it is noteworthy that the possibility of lymphoma needs to be excluded by immunohistochemistry if the uniform size of lymph cells and nuclear irregularities are noticed [107].

## Treatment strategies

The treatment approach for SMCL is profoundly influenced by the concomitant coexistence or not of an overt or classic MCL (Table 1). For patients without evidence of overt lymphoma, a “watch and wait” strategy is strongly suggested and overtreatment may be avoided [47]. Compared with other indolent lymphoma, the more aggressive behavior of overt MCL could suggest a closer follow-up. A follow-up strategy include evaluation every 3 months (at least 6 months) for MIPI scores, imaging study, morphology and pathological biopsies. However, due to the feasibility of clinical practice, imaging evaluation may only be performed in the presence of disease-related symptoms or organ involvement. Meanwhile, if novel nodal or extranodal lesion is found, biopsy is mandatory to exclude lymphoma. Generally, there are no indications to start treatment in “in situ” MCL patients, without clear evidence of concomitant or subsequent overt MCL.

However, early detection and treatment may improve the prognosis of MCL in the event of high risk mutation, such as *C-myc, TP53* and *NOTCH1/2* mutation, etc., and the transformation of histotype, progression of stage and localization of overt lymphoma.

## Conclusions

We coined the term “SMCL” for the asymptomatic MCL cases with indolent clinical behavior. Our proposed definition of SMCL is: lack of B symptoms, normal serum LDH and b2-microglobulin levels, low MIPI score, maximum tumor diameter less than 3 cm, spleen size < 20 cm, PET/CT with the SUVmax <6, Ki-67 less than 30%, with some particular immunophenotype, such as CD5 and CD38 negative, markedly increased CD23 positive lymphocytes proportions (typical MCL usually has negative CD23), high expression of CD200, kappa light chain restriction (Typical MCL usually has lambda light chain restriction), without *C-myc, TP53* and *NOTCH1/2* mutation, nonblastoid/pleomorphic histology, and no tumor growth on reevaluation every 3 months, at least 6 months. The common clinical and pathological forms of SMCL may include MBL with t (11;14), nonnodal leukemic MCL and in situ lymphoma (including GI in situ lymphoma).

These clinical and pathological forms remain stable or grow very slowly over a long period of time without any symptoms or with only mild clinical symptom. It may take up to 12~15 years for SMCL progress to classic MCL. Thus, unnecessary treatment which can bring potential harm to patients can be avoided. Importantly, close observation is still needed. Some SMCL may need early intervention in order to avoid transformation to classic MCL. In addition, the mechanism of transformation from SMCL to classic MCL is not very clear, which may be partly due to additional genetic mutations which include *17p/TP53, NOTCH1/2*, *C-myc*, or other gene mutations.

## Abbreviations

ATM: Ataxia-telangiectasia mutated
CHK2: Cell cycle checkpoint kinase 2
CLL: Chronic lymphocytic leukemia
EFS: Event-free survival
FFPE: Formalin-fixed paraffin-embedded
GI: Gastrointestinal
IgH: Ig heavy chain
LDH: Lactic dehydrogenase
MBL: Monoclonal B-lymphocytosis
MCL: Mantle cell lymphoma
MIPI: Low MCL-International Prognostic Index
MLP: Multiple lymphomatous polyposis
OS: Overall survival
PET/CT: Positron Emission Tomography/computerized tomography
SMCL: Smoldering mantle cell lymphoma
SUV: Standard Uptake Value
WBC: White blood cell
WHO: World Health Organization
β2M: β2-microglobulin levels
